# A review of total & added sugar intakes and dietary sources in Europe

**DOI:** 10.1186/s12937-016-0225-2

**Published:** 2017-01-21

**Authors:** Véronique Azaïs-Braesco, Diewertje Sluik, Matthieu Maillot, Frans Kok, Luis A. Moreno

**Affiliations:** 1 VAB-Nutrition, 1, rue Claude Danziger, 63100 Clermont-Ferrand, France; 2 Division of Human Nutrition, Wageningen University and Research, Wageningen, The Netherlands; 3 MS-Nutrition, 27, BD Jean Moulin, 13005 Marseille, France; 4 GENUD (Growth, Exercise, Nutrition and Development) Research Group, Instituto Agroalimentario de Aragón (IA2), Instituto de Investigación Sanitaria Aragón (IIS Aragón), Centro de Investigación Biomédica en Red Fisiopatología de la Obesidad y Nutrición (CIBERObn), University of Zaragoza, Zaragoza, Spain

**Keywords:** Added sugars, Contribution of food categories, National dietary surveys, Socio-economic status

## Abstract

Public health policies, including in Europe, are considering measures and recommendations to limit the intake of added or free sugars. For such policies to be efficient and monitored, a precise knowledge of the current situation regarding sugar intake in Europe is needed. This review summarizes published or re-analyzed data from 11 representative surveys in Belgium, France, Denmark, Hungary, Ireland, Italy, Norway, The Netherlands, Spain and the UK. Relative intakes were higher in children than in adults: total sugars ranged between 15 and 21% of energy intake in adults and between 16 and 26% in children. Added sugars (or non-milk extrinsic sugars (NMES), in the UK) contributed 7 to 11% of total energy intake in adults and represented a higher proportion of children’s energy intake (11 to 17%). Educational level did not significantly affect intakes of total or added sugars in France and the Netherlands. Sweet products (e.g. confectionery, chocolates, cakes and biscuits, sugar, and jam) were major contributors to total sugars intake in all countries, genders and age groups, followed by fruits, beverages and dairy products. Fruits contributed more and beverages contributed less to adults’ total sugars intakes than to children’s. Added sugars were provided mostly by sweet products (36 to 61% in adults and 40 to 50% in children), followed by beverages (12 to 31% in adults and 20 to 34% in children, fruit juices excluded), then by dairy products (4 to 15% in adults and 6 to 18% in children). Caution is needed, however, as survey methodologies differ on important items such as dietary data collection, food composition tables or estimation of added sugars. Cross-country comparisons are thus not meaningful and overall information might thus not be robust enough to provide a solid basis for implementation of policy measures. Data nevertheless confirm that intakes of total and added sugars are high in the European countries considered, especially in children, and point to sweet products and beverages as the major contributors to added sugar intakes.

## Introduction

The role of excessive sugar intake on health and disease is currently an active area of scientific and policy debate. Following a direction clearly indicated by WHO guidelines [1], many countries are today considering regulations or public health policy measures aiming at lowering sugar intakes in their population, and especially in children [2]. These concerns are justified by studies and reports indicating that high intakes of sugars are associated with an increased risk of dental caries [3], overweight [4] and cardio-metabolic risk factors and mortality [5, 6]. However, uncertainty and controversies remain as to whether sugar intake is directly related to these health outcomes or whether they are rather due to an excessive energy intake [7, 8]. For example, the change in body adiposity that occurs with modifying sugar intakes seems to be mediated via changes in energy intakes, since isoenergetic exchange of sugars with other carbohydrates is not associated with a difference in weight change [4]. By contrast, sugar intake influences blood pressure and serum lipids, independently of the effect of sugars on body weight [5]. In any case, decreasing sugar consumption is a good strategy to lower excessive energy intake, which is relevant to the current obesity epidemic.

Across countries, several different recommendations address the intake of total, added, or “free” sugars. Free sugars are defined as “monosaccharides and disaccharides added to foods and beverages by the manufacturer, cook or consumer, and sugars naturally present in honey, syrups, fruit juices and fruit juice concentrates” by WHO, which recommends reducing their intake to less than 10% of total energy intake for children and adults [1]. The American Academy of Pediatrics recommends that children use “the minimum amount of added sugar necessary to promote the palatability, enjoyment, and consumption of nutrient-rich food items” [9]. The 2015 edition of the Dietary Guidelines for Americans also adopts a 10% threshold for added sugars [10], and the amount of added sugar will be labeled on US food packages in 2019 at the latest [11]. The European situation is today contrasted: the 10% limit has been highlighted in the Nordic Nutrition Recommendations since 2014 [12] and the UK has adopted in 2015 an even more demanding threshold, with a recommended intake of less than 5% of dietary energy as free sugars [13]. Most other countries have not set quantitative reference intakes, but guidelines may mention that high intakes may be detrimental to nutrition and health, for instance 20% of the energy as added sugars in the Netherlands [14].

In order to promote dietary patterns fitting with the current recommendations on free sugars consumption, the first step should be to know the current intake of total, added and free sugars, the adherence to the recommendations and the main food sources for sugars consumption. Because obesity and diet-related diseases are especially worrying in populations with lower incomes and educational levels [15], they should be a priority target and knowledge on sugar intakes in this population is warranted. While intakes of sugars have recently been reviewed worldwide [16], there is scarce information concerning the main food sources of sugars and the role of education and income levels. Scattered information has been published, such as in Australia, where sugar-sweetened beverages accounted for the greatest proportion of sugars consumption, followed by sugar and sweet spreads, and by cakes, biscuits and pastries [17]. In Canada, the major source of added sugars was confectionery in children aged 1 to 8, and soft drinks in older children, teens and adults [18].

In echo to the discussions currently occurring at the European level [19], the present review aims to summarize and review the available data from representative nation-wide surveys in the European Member States concerning the various characteristics of sugar intakes in children and adults, with the aim of informing stakeholders and policymakers.

## Material & methods

### Definition of sugars

From a biochemical point of view, sugars correspond to dietary monosaccharides, i.e. glucose, fructose, and galactose, and disaccharides, i.e. sucrose and lactose.

Distinctions are made between “total sugars”, encompassing all naturally occurring sugars and “added sugars”, corresponding to those added to foods by the manufacturer, cook or consumer. Several ingredients can be used for this purpose, including sucrose, fructose, glucose, high fructose syrup or concentrated fruit juices, some of them also being naturally present in foods. Some surveys report the intake of sucrose as the sole information about sugar intakes. However, sucrose should neither be confused with added sugars nor with total sugars, as several other sugars can be added to foods and as sucrose is naturally present in foods such as fruits. A third concept, referring to “free sugars”, defined as “added sugars plus sugars naturally present in honey, syrup and fruit juices” has been defined by WHO [1]. In the UK, a slightly different entity has been used in the survey analyzed in this report, named “non-milk extrinsic sugars” (NMES), which corresponds to sugars not contained within the cellular structure of a food, except lactose in milk and milk products. The difference between NMES and free sugars is that non-milk extrinsic sugars include 50% of the fruit sugars from stewed, dried or canned fruit (assuming that processing changes intrinsic sugars into extrinsic ones) but free sugars does not take processing effects into account [20]. In this review, data have been gathered on total sugars, added sugars and NMES; only one survey reported intakes on “free sugars” [21]. In some studies analyzed in this review, the wording “soluble carbohydrates” can be found, and this term was assumed to correspond to “sugars” [22].

### Survey selection

Our purpose was to identify representative nation-wide surveys, which have been systematically searched for on Medline, using the key words [sugar AND (intake or diet or survey)] associated to the name of each of the EU 28 countries, plus Switzerland and Norway. This retrieved 107 hits, with one of them only fitting to our criteria. A hand search was thus undertaken on Google, and on the websites of national Public Health Authorities or Agencies in European countries. For the purpose of this review only country-representative surveys carried out in Europe and which reported exploitable data on sugar intakes were selected. Selection criteria and study eligibility were agreed among all authors. When several surveys were identified in the same country, only the most recent was analyzed and no survey older than 20 years was retained since the aim was to present the most current intakes. Eleven nation-wide surveys, providing reliable data on total and/or added sugars or NMES have been identified in 10 countries: Belgium [23], Denmark [24], France [25], Hungary [26], Ireland [27, 28], Italy [22, 29], the Netherlands [21, 30], Norway [31], Spain [32, 33], and the UK [34] (Table 1). Information has been also identified from representative surveys carried out in Austria [35], Finland [36] and Germany [37, 38]. However, the Austrian and Finnish reports provided sugar data for sucrose only, and the German survey reported separately figures for monosaccharides and disaccharides. As these data were not in a format consistent with those of the other surveys, they were not included in the tables or figures, but were considered in the results and discussion.

**Table 1:** Characteristics of eleven included European nation-wide surveys

Country	Year of survey	Adults				Children-adolescents				Dietary data collection method
		Sugar type	Age range	Sample size		Sugar type	Age range	Sample size		
				Female	Male			Female	Male	
Belgium [23]	2004	Total	19 to 59	641	675	Total	15 to 18	608	780	24 h recall + FFQ
Denmark [24]	2011–2013	Added	18 to 75	1552	1464	Added	10 to 17	258	251	7-day record
France (re-analyzed from [25])	2007	Total & added	18 to79	994	902	Total & added	3 to 17	700	745	7-day record
Hungary [26]	2009	Added	18 to 100	1717		Not available				3-day record
Ireland [27, 28]	1997–1999	Total & added	18 to 64	1379		Total & added	13 to 17	441		7-day record
Italy [22, 29]	2005–2006	Total	18 to 65	1245	1068	Total	10 to 18	139	108	3-day record
Norway [31]	2010–2011	Added	18 to 70	925	862	Not available				2 24-h recalls
The Netherlands (re-analyzed from [30])	2007–2010	Total & added	19 to 69	1050	1054	Total & added	7 to 18	857	856	2 24-h recalls
Spain [32, 33]	2013	Total	18 to 64	857	798	Total	13 to 17	74	137	3-day record
United Kingdom [34]	2008–2012	Total & NMES	19 to 65	1571	1126	Total & NMES	4 to 18	1365	1409	4-day record

### Survey designs

The identified surveys differed by several parameters, such as the age range of the considered populations, the dietary data collection methods or the year of the field survey (Table 1). Random sampling was performed to ensure the best possible representativeness of the population of the country, using most often electoral registers [39], census information or phone books, followed by appropriate weighting for socio-demographic parameters, such as in France, the Netherlands, the UK, and Germany [25, 30, 34, 37], but not in Denmark [24]. While most surveys were reported as having been carried out over the four seasons of the year and have recorded data on week and week-end days, statistical adjustment for seasonality and week of the day have only been performed in France and the Netherlands [25, 30]. Under-reporting or over-reporting subjects were identified in many surveys [23, 25–27, 30, 37, 40]. Extreme reporters were excluded in most cases, but not in Norway [31], the Netherlands [30] or Italy [40] and, in Ireland, only for adults but not children [27]. In Denmark and Germany, exclusion of extreme reporters was not clearly acknowledged [24, 37]. We had access to both the raw dataset from the Dutch [30] and the French [25] dietary surveys, which have been re-analyzed specifically for this review when information was missing from the reports or publications.

### Dietary assessment

Dietary data collection was carried out with different tools, developed specifically in each country, except in the Netherlands and in Belgium, which both used the EPIC-software [41]. As a consequence, the coding of the recorded food items varies across countries, which resulted in different classifications of foods within groups, categories and subcategories. Harmonized food categories, i.e. containing similar food sub-groups, have been elaborated by redistributing food sub-groups in a consistent way, when possible and as shown in Table 2. Only categories that contribute significantly to sugar intakes were considered. In the Irish surveys, contributions of added sugars were given but these surveys omitted some important categories, such as processed fruits or dairy products; these data have not been included in tables and figures [27].

**Table 2:** Food items taken into account in harmonized food groups according to countries

Country	Fruits & vegetable	Dairy products	Sweet products	Beverages
Belgium [23, 53]	Pit fruits, fruits unclassified, vegetables	Milk; yoghurts; cream desserts/pudding (milk-based)	Cakes/pies/pastries/puddings; dry cakes/biscuits; sugar/honey/jam; chocolate(products)	Carbonated/soft/isotonic drinks; fruit & vegetable juices; alcoholic drinks
France [25]	Fruits, cooked fruits and fruit sauces; vegetables (except potatoes)	Milk; fresh dairy products (yoghurt and fresh cheese); cheese; dairy desserts/cream desserts/gelled milks	Viennoiseries (croissants, etc.); sweet and savory biscuits and bars; cakes & pastries; ice creams & frozen desserts; sugars and candies; chocolate	Fruit & vegetable juices; fruit nectars; soft drinks; coffee; other hot beverages; alcoholic drinks
Italy [29]	Fruits, fresh and processed; vegetables, fresh and processed; spices and herbs	Milk/milk-based beverages; yoghurts/fermented milks; cheese; milk-based desserts & substitutes	Biscuits; savory fine bakery products; cakes & sweet snacks; ice cream/ice lollies and substitutes; chocolate & substitutes; candies, jam & other sweet products; cocoa & cocoa-based powder	Coffee, tea, herbal tea and substitutes; fruit & vegetable juices; other soft drinks: alcoholic beverages & substitutes
Norway [31]	Vegetables, fruits, berries, jams, preserved fruits; nuts, olives, seeds	Milk (all kinds), yoghurt, cheese	Sugars and sweets; cakes	Pure fruit juices; soft drinks and fruit drinks; beer, wine; liquor
Spain [33]	Fruits; vegetables	Milks, cheeses, yoghurt & fermented milks; other dairy products	Bakery & pastries; sugar; chocolates; jams & others; other sweets	Coffee & infusions; sugary soft drinks; non-sweetened soft drinks; sports drinks; energy drinks; juices & nectars; other drinks; alcoholic beverages
The Netherlands [21]	Fruits, nuts& olives/vegetables	Milk; dairy beverages; yoghurt; cottage cheese; coffee creamer	Sugar/honey/jams; confectionery; chocolate; syrups; ice creams; cake & cookies	Fruit & vegetable juices; soft drinks; coffee/tea; alcoholic beverages
United Kingdom [34]	Fruits; nuts and seeds; vegetables& potatoes	Milk and milk products (excluding ice cream)	Sugar/preserves& confectionery (including chocolate); ice creams; biscuits; buns/cakes/pastries & fruit pies	Fruit juices; soft drinks; tea/coffee; alcoholic beverages; dry weight beverages

### Assessment of added sugars and NMES

Also of relevance is the way the intake in added sugars or in NMES has been estimated. To our knowledge, no national food composition database currently contains values for added or free sugars. The method used to estimate the content of added sugar was not clearly detailed in Hungary and Norway [26, 31]. In France, Ireland, the Netherlands and the UK [21, 25, 28, 34], the added sugars content was approached using disaggregated recipes, either as they existed in the national food composition database or defined from cookbook or manufacturer’s information, and/or using the ingredient list or other piece of information from the labeling. Naturally occurring sugars from fruits, vegetables and milk were not included. In Denmark [24], the whole sugar in specific food groups, such as sweets, cakes, soft drinks, desserts, breakfast cereals were considered as added sugars.

### Statistical analysis

Because of significant differences in the surveys’ methodologies, it would be inappropriate to comment on the differences observed across countries, but some trends can be identified, which have not been statistically treated. When assessing the relationship between the educational level and sugar intakes (Table 5), original data from the French and the Dutch surveys were re-analyzed and the educational levels were defined as follows: for the Netherlands, ‘low’ was assigned to primary and lower vocational education,’intermediate’ to advanced elementary, intermediate vocational and higher general secondary education and ‘high’ to university or higher vocational education; for France, ‘low’ was assigned to mid-secondary or below, ‘intermediate’ to high school and ‘high’ to university education. Total and added sugar intakes across levels of education were calculated using general linear models. Models were adjusted for age (years), sex, and energy (kcal/day). A P-value for trend was calculated with a contrast statement.

## Results

### Intake in total sugars

Table 3 displays an overview of the intakes in total sugars in adults and children, for both genders, in absolute values (g/day) as well as a percentage of the daily energy. In all countries and at all ages, women/girls had a lower intake in sugars than men, when expressed in g/day, but this difference disappeared when the sugar contribution to the total energy intake was considered, likely reflecting the higher energy intake of males. In adults, sugar contributed more to women’s than to men’s energy intake (8 to 17% more, except in the UK: +3.5%). This result is confirmed in Germany, where the estimated energy contribution of sugar (sum of mono- and disaccharides, data not shown) was 19.3 and 24.0% in men and women respectively, aged 15 to 80 [37].

**Table 3:** Total sugar intakes and their contribution to energy in selected European countries

Country & year of survey	Adults						Children					
	Total sugar intake (g/day)-mean ± SD			Total sugar contribution to daily energy intake (%)- mean ± SD#			Total sugar intake (g/day)-mean ± SD			Total sugar contribution to daily energy intake (%)- mean ± SD#		
	Female	Male	Both	Female	Male	Both	Girls	Boys	Both	Girls	Boys	Both
Belgium, 2004. ([23])	97.1*	132.5*	115.3*	20.9 ± 6.2	19.1 ± 5.4	19.9 ± 5.8	121.5*	180.1*	150.3*	23.9 ± 5.7	25 ± 7.3	24.5 ± 6.6
France, 2007 (retreated from [25])	84.8 ± 31.2	97.9 ± 46.1	91.1 ± 38.5	17.6.0 ± 4.9	15.0 ± 5.6	16.41 ± 5.4	87.2 ± 29.2	99.7 ± 34.9	93.6 ± 32.6	20.8 ± 4.7	20.6 ± 5.0	20.7 ± 4.8
Ireland, 1997–99 [27, 28]	Not available		108.3 ± 44.8	Not available		16.8 ± 4.8	Not available		108.5 ± 43.0	Not available		20.4 ± 5.0
Italy, 2005–06. [22]	79.5 ± 33.4	86.0 ± 37.7	82.5**	15.4 ± 5.1	13.5 ± 4.7	14.5**	88.4 ± 35.6	107.6 ± 53.7	96.8**	15.8 ± 5.2	15.4 ± 4.7	15.6**
The Netherlands, 2007–10 (retreated from [30])	108.3 ± 47.4	125.5 ± 60.5	116.9 ± 55.4	21.3 ± 6.8	19.7 ± 7.3	20.5 ± 7.1	133.9 ± 23.5	151.9 ± 28.3	143.1 ± 26.5	25.9 ± 3.5	25.8 ± 3.6	25.8 ± 3.5
Spain, 2013 [32, 33]	72.4*	78.6*	75.8*	17.3	16	16.7	87.5*	89.7*	89.3*	19.2	16.9	17.7
United Kingdom, 2008–12 [34]	84.6 ± 39.4	105.6 ± 48.3	93.4**	20.5 ± 6.8	19.8 ± 6.6	20.2**	92.3 ± 34.6	108.8 ± 42.4	100.7**	22.6 ± 6.3	22.7 ± 6.2	22.7**

*SD* Standard deviation- This table reports values as reported in source documents or as re-treated from individual raw data, except for those with asterisks, which should be taken as indicative only. Contribution to daily energy intake considers energy from alcohol, except for Belgium data

*these values have been calculated from mean values of energy and sugar intakes, as reported in source documents

**these values combining both gender have been calculated from the values and sample size for each gender

#SD not available on Spanish data

In children, this trend is much weaker and often does not exist, which is consistent with the absence of a gender difference in total energy intake in younger children. Of relevance is also the higher intake and energy contribution of sugars in children as compared with adults. This was observed to a large extent in Belgium, where sugar intakes were 30% higher in children, or in the Netherlands (22% higher), but less so in the UK (8% higher). In Belgium, 80.1% of the adult population and 94.6% of the child population was exceeding 15% of energy provided by total sugars [23].

In all surveys, except those from France [25] and Norway [31], data were also available on more detailed age ranges, in addition to those displayed in Table 1. Available data suggest that the contribution of sugar to energy was slightly higher in younger children than in older ones; in Ireland, this contribution is of 23.9 ± 5.3% in children aged 5 to 12, vs 20.4 ± 5.0 in the 13 to 17 age group [27] and similar observations can be made in the UK ([34]; data not shown). In adults, there might be a trend toward a slight decrease in the energy provided by sugars with increasing age. In Belgium, total sugar is 19.9 ± 5.8% of the energy intake in the group aged 19 to 59, 19.1 ± 5.3% in people aged 60 to 74 and 18.7 ± 6.1% in those above 75 [23]. However, this trend is not observed in the older groups in other countries: in the UK or in Spain, the elderly above 65 have respectively 1.8 or 1.6% more energy from sugar than the adults aged 19 to 64 [32, 34].

### Intake in added sugars or NMES

Table 4 displays an overview of the intakes in added sugars or NMES (UK) in adults and children, for both genders, in absolute values (g/day) and as a percentage of the daily energy. The same gender differences as for total sugars were seen for added sugars, in each country, with a higher intake in men (+14% in Hungary [26] and up to +49% in the UK [34]). The contribution of added sugars to the energy intake was not dependent on gender, in adults or in children. Conversely, there is a strong age effect: added sugar contributes at least 30% more to total energy intake in children vs adults (from + 32% in Ireland [28] up to + 50% in the Netherlands [30]).

**Table 4:** Intake of added or non-milk extrinsic sugars and contribution to energy in selected European countries

Country	Adults						Children					
	Added or NME sugar intake (g/day)-mean ± SD			Added sugar contribution to daily energy intake (%)-mean ± SD			Added or NME sugar intake (g/day)-mean ± SD			Added sugar contribution to daily energy intake (%)-mean ± SD		
Adults	Female	Male	Both	Female	Male	Both	Girls	Boys	Both	Girls	Boys	Both
Denmark [24]	43.0 ± 30.2	56.0 ± 44.6	49.0 ± 38.4	8 ± 5.0	8 ± 5.4	8 ± 5.2	53 ± 34.4	67 ± 34.3	60 ± 35.1	11.0 ± 5.0	11.0 ± 5.0	11.0 ± 5.0
France, (retreated from [25])	41.6 ± 25.6	49.6 ± 38.5	45.4 ± 31.7	8.5 ± 4.5	7.5 ± 5.1	8.0 ± 4.8	50.3 ± 21.8	62.6 ± 29.3	57.1 ± 26.2	12.2 ± 4.12	12.9 ± 4.7	12.5 ± 4.4
Hungary [26]	44.0 ± 26.2	50.2 ± 35.3	46.1 ± 30.7	8.2 ± 4.3	7.0 ± 4.4	7.6 ± 4.4	Not available					
Ireland [27, 28]	Not available		61,9 ± 3 7,7	Not available		9,4 ± 4,3	Not available		65,7 ± 31,6	Not available		12,4 ± 4,9
Norway [31]	36 ± 30	48 ± 43	42 ± 38	7,4 ± 5,2	7,2 ± 5,7	7,3 ± 5,4	Not available					
The Netherlands (retreated from [30])	68.2 ± 44.3	83. 9 ± 55.9	76 ± 51.3	11.1 ± 6.4	11.3 ± 6.4	11.2 ± 6.6	98.2 ± 21.3	113.7 ± 26.8	106 ± 24.7	17.1 ± 3.5	16.5 ± 3.3	16.8 ± 3.4
United Kingdom [34]	41.6 ± 35.0	62.2 ± 41.8	50.2*	11.1 ± 6.3	11.9 ± 6.0	11.4*	61.5 ± 30.4	74.6 ± 38.6	68.1*	14.8 ± 5.8	15.4 ± 6.1	15.1*

This table reports values as reported in source documents or as re-treated from individual raw date, except for those with an asterisk (*), which combine both gender and have been calculated from the values and sample size for each gender. Contribution to daily energy intake considers energy from alcohol

Austrian and Finnish national surveys report data on sucrose intakes only, which were in the same order of magnitude as the added sugars. In Austria, adults aged 25 to 50 y received 9% of their energy intake from sucrose, girls and boys aged 13–14 received respectively 11 and 10% [35]. In Finnish adults aged 24–64 y, men consumed daily 53.5 ± 37.1 g of sucrose (9.7 ± 5.9% of energy) vs 42.9 ± 26.3 g (10.5 ± 5.1% of energy) in women [36].

In the Netherlands, 45 and 10% of the adults and children, respectively, received less than 10% of their energy as added sugars [21]. In the UK, the median intake of NMES in adults is 10.6% of energy, indicating that half of the adult population had a higher intake and that many more were exceeding the 5% UK threshold for free sugars. In children, the median NMES intake was above 14%, suggesting that a larger proportion than in the adults’ population was exceeding recommended thresholds (data not shown) [34].

### Educational level

In Table 5, intakes of total and added sugar are displayed according to educational level in the Netherlands and France, adjusted for age, sex, and energy intake. In the Netherlands, total sugar intake did not differ greatly according to educational level. Added sugar intake was significantly lower with a higher educational level, both in adolescents and adults. In France, total sugar intake was lower in children and adolescents with a higher educational level vs a lower one, but the trend seems in the opposite direction for adults. However, for total as well as for added sugar, no significant trend can be detected.

**Table 5:** Adjusted intakes^a^ of total and added sugar according to education level in adults and teenagers

Country	Age range	Education level (n)	Sugar intake (g/day) - mean ± SE	
			Total sugars	Added sugars
The Netherlands [30]	12 to 18 years	Low (228)	144.8 ± 2.4	99.2 ± 2.3
		Intermediate (681)	144.3 ± 1.3	93.7 ± 1.2
		High (72)	135.5 ± 4.4	84.5 ± 4.2
		P-trend	0.08	0.004
	19 to 69 years	Low (329)	117.8 ± 1.9	70.3 ± 1.8
		Intermediate (1312)	118.0 ± 0.9	68.0 ± 0.9
		High (463)	118.3 ± 1.5	62.0 ± 1.4
		P-trend	0.83	0.0002
France [25]	3 to 17 years	Low (194)	98.2 ± 2.5	62.8 ± 2.8
		Intermediate (778)	92.1 ± 1.0	56.7 ± 0.8
		High (438)	93.9 ± 1.0	54.6 ± 1.0
		P-trend	0.1924	0.0830
	18 to 79 years	Low (338)	86.4 ± 2.4	45.5 ± 2.1
		Intermediate (978)	88.6 ± 1.0	43.9 ± 0.8
		High (546)	90.2 ± 1.4	42.4 ± 1.2
		P-trend	0.4087	0.3146

^a^Adjusted for age (years), sex (male/female), and energy (kcal/day)

### Contributors to intake of total sugars

**Fig. 1:**
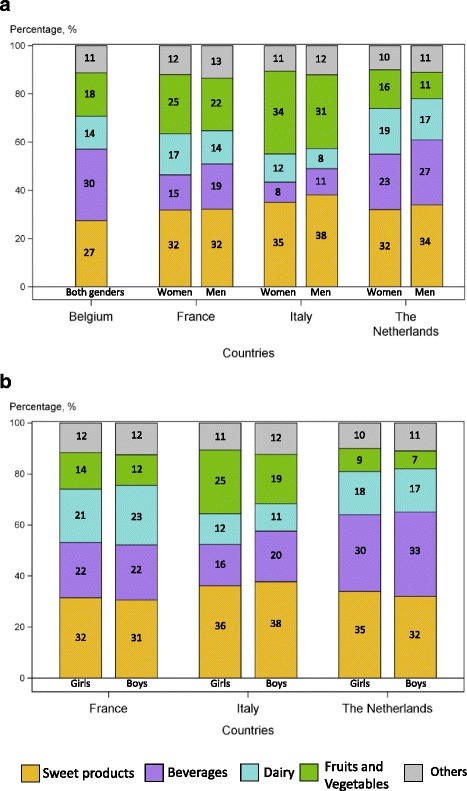
**a**. Contributors to total sugars among adults. **b**. Contributors to total sugars among children

Contributions of different food groups to intakes of total sugars are displayed in Fig. 1 and Table 6. Sweet products (Table 2) were major contributors to the intake of total sugars in all countries and across genders and ages. The other important contributors were fruits and vegetables, beverages and dairy products, with a ranking which may vary according to geographies and ages. Fruits and vegetables were major contributors (more than 20% of total sugar intakes) in Southern European adults (Spain, Italy and France), but less so in the Netherlands or Belgium (11 to 18%). In children, this high level of contribution of fruits and vegetables to intake of total sugars remained in Italy only.

**Table 6:** Percentage contribution of selected food groups to the intake of total sugars in adults and children^a^

Country		Adults								Children						
		Belgium [23, 53]	France (adapted from [25])		Italy [22, 29]		Spain [32, 33]	The Netherlands (adapted from [30])		France (adapted from [25])		Italy [22, 29]		Spain [32, 33]	The Netherlands (adapted from [30])	
Age		>15	18 to 74		18–65		18–64	19 to 69		3 to 17		10 to 18		13 to 17	7 to 18	
Gender		Both	Women	Men	Women	Men	Both	Women	Men	Girls	Boys	Girls	Boys	Both	Girls	Boys
Dairy	Total dairy	14*	17	14	12	8	23	19	17	21	23	12	11	24	18	17
	* Milk, dairy beverages*	4	5	4	7	6	12	9	9	9	9	9	9	13	10	8
	* Dairy desserts*	3	4	4	1	0	3	3	3	5	6	0	0	5	2	3
	* Yoghurt*	4	8	6	3	1	7	6	5	7	8	2	1	6	6	6
	* Cheese*	NA	0	0	1	1	1	1	0	0	0	1	1	0	0	0
Sweet products	Total sweet	27	32	32	35	38	21	32	34	32	31	36	38	24	35	32
	* Cake & cookies*	11	12	11	9	8	7	12	10	15	15	13	14	8	10	9
	* Syrups, sugar, honey, jam*	10	15	17	21	23	10	10	13	7	7	12	13	4	9	9
	* Confectionary*	6			2	2	0	3	3			1	1	1	5	5
	* Chocolate*		3	2	1	2	4	5	6	7	6	6	5	11	8	7
	* Ice cream*	NA	2	2	2	3	NA	2	2	3	3	4	5	NA	3	2
Beverages	Total Bev.	30	15	19	8	11	23	23	27	21	22	16	20	29	30	33
	* F&V juices*	6	6	5	3	3	6	9	7	9	7	9	9	12	9	8
	* Soft drinks*	19	5	7	3	4	11	11	16	9	11	5	8	17	21	25
	* Hot beverages*	NA	2	3	1	1	2	1	2	4	4	2	3	0	0	0
	* Alcoholic bev*	5	2	4	1	3	4	2	2	0	0	0	0	0	0	0
Fruits & vegetables		18	25	22	34	31	24	16	11	14	12	25	19	12	9	7
Others		11	12	13	11	12	9	10	11	11	12	11	12	11	10	11

^a^In % of daily intake of added sugars- NA: not available- See text for details

*total is higher that the sum of dairy items, as reported in the quoted source

In all countries, beverages contributed more to intakes of total sugars in children than in adults (+15 to +30% in children vs adults) and in Italy sugars from beverages contributed twice as much to children’s energy intake than to adults’. Soft drinks provided more sugars than fruit juices in most countries and most age ranges, especially in the Netherlands, Belgium and Spain, whereas in Italy and France, sugars intakes from beverages were lower and came nearly equally from juices and soft drinks. Overall, the dairy contribution to total sugar intakes was the lowest in Italy and the highest in Spain and France. Milk and dairy beverages were the major contributors within dairy products, especially in children.

### Contributors to intake in added sugars and NMES

**Fig. 2:**
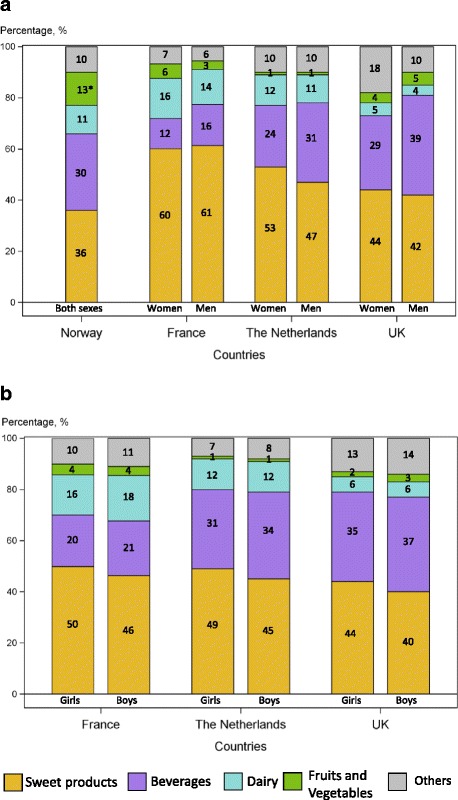
**a**. Contributors to added-sugars among adults. **b**. Contributors to added-sugars among children

Figure 2 and Table 7 display the available information regarding the food sources of added sugars or NMES. Sweet products provided 47 to 61% of those in adults and 40 to 50% in children. Beverages were the second highest contributor to added sugars, except in French adult women. They provided 12 to 31% of those in adults and 20 to 37% in children and most of this contribution came from soft drinks, with 0 to 5% of added sugars coming from fruit nectars (i.e. drinks which contain at least 55% of fruit juices and to which sugar can be added). When NMES are considered rather than added sugars, fruit juices became significant contributors, which translated into a higher overall contribution of beverages in the UK, compared with other countries. In adults, alcoholic beverages had a small, but real contribution to adults’ intakes of added sugars or NMES. Irish [27] and Danish [24] data, although using a different definition of food categories were, however, globally consistent with this picture, with sweet products as first contributors, followed by soft drinks (data not shown).

**Table 7:** Percent contribution of selected food groups to the intake of added or NME sugars in adults and children^a^

		Adults						Children					
Country		The Netherlands (adapted from [30])		France (adapted from [25])		UK [34]		The Netherlands (adapted from [30])		France (adapted from [25])		UK [34]	
Type of sugars		Added		Added		NMES		Added		Added		NMES	
Age		19 to 69		18 to 79		19 to 64		7 to 18		3 to 17		4 to 18	
Gender		Women	Men	Women	Men	Women	Men	Girls	Boys	Girls	Boys	Girls	Boys
Food group	*Category*												
Dairy	Total dairy	12	11	16	14	5	4	12	12	16	18	6	6
	* Milk & dairy beverages*	3	2	0	0	1	1	4	3	0	0	2	2
	* Dairy desserts*	4	4	8	8	4	3	3	3	8	9	4	4
	* Yoghurt*	4	4	8	6			5	5	8	9		
	* Cheese*	1	1	0	0	NA	NA	0	1	0	0		
Sweet products	Total sweet products	53	47	60	61	44	42	49	45	50	46	44	40
	* Cake & cookies*	16	12	23	20	17	13	12	10	24	22	18	16
	* Sugar, honey, jam, syrup*	17	18	27	32	16	19	12	13	11	11	8	8
	* Confectionary*	6	5			2	1	9	8			6	6
	* Chocolate*	10	9	5	5	7	7	11	10	10	9	8	7
	* Ice cream*	4	3	5	4	2	2	5	4	5	4	4	3
Beverages	Total beverage	24	31	12	16	29	39	31	34	20	21	35	37
	* F&V juices*	3	3	1	0	8	8	5	4	0	0	11	12
	* Soft drinks*	18	26	7	10	15	16	26	30	13	15	22	24
	* Coffee, tea*	1	1	2	3	0	1	0	0	7	6	1	0
	* Alcoholic beverages*	2	1	2	3	6	14	0	0	0	0	1	1
Fruits & vegetables		1	1	6	3	4	5	1	1	4	4	2	3
Others		10	10	7	6	18	10	7	8	10	11	13	14

^a^In % of daily intake of added sugars- NA: not available- See text for details

The contribution of dairy products to added sugars or NMES was between 4 and 16% in adults and between 6 and 18% in children, the highest contribution being observed in France. Dairy beverages provided 20% to a third of dairy added sugars, except in France where dairy beverages, consumed mostly at home by children are included in the “hot beverages” section. Yoghurt and dairy desserts contributed roughly equally.

## Discussion

This review has focused on nation-wide representative studies carried out in Europe where exploitable information about total sugar intake was found for 13 countries, i.e. Denmark, France, Hungary, Ireland, The Netherlands, Norway, the UK, Austria, Belgium, Finland, Germany, Italy and Spain. Only the first seven of these surveys provided estimates on added sugar intake.

While differences in methodological surveys prevent direct and statistical comparisons between countries, some relevant conclusions can be deduced from the data, which may inform initial policy steps by identifying extent of excess in sugar intakes as well as the major contributors in the concerned age ranges. Sugars have a significant contribution to total energy intake in all countries, genders and age groups, with approximate ranges of 14.5 to 20.5% in adults and 15.6 to 25.6% in children. This difference between adults and children appears even larger when added sugars or NMES are considered, which contribute 7.3 to 11.4% to energy intake in adults and 11.0% to almost 16.8% in children. A higher sugar intake in children vs adults is not a recent feature: in the first NHANES (National Health and Nutrition Survey) survey in the USA, in 1971–1975, total sugar was already contributing 22% more to children’s energy intake than to adult’s. This age difference was less important (10%) 17 years later [42].

The ratio of added sugars or NMES over total sugar is always higher in children than it is in adults, in the studies we have identified. This is especially clear in France, where 49.8 and 64.4% of the sugars consumed by adults and by children respectively are added sugars, but this trend can be also seen in Ireland, UK and the Netherlands. A French survey, different from the one reported in Table 7, found that 44.8% of children aged 3 to 17, but 73.9% of adults, received less than 12.5% of their energy from sugars coming from sweet products, a group that comprised honey, jam, chocolate and confectionery, cakes, pastries, biscuits, desserts, fruit juices, soft drinks, and breakfast cereals [43]. It is unclear if this difference is due to a generational effect, with children being more prone to sweet/sweetened products than adults, or to a trend to consume more added sugars, which might lead children to maintain high intakes when they become adults. This difference might also be cultural, as it is not observed in the USA, where added sugars represent around 65% of total sugars in both age ranges, with no changes between 1971 and 1988 [42].

Age differences can also be noticed when addressing the food groups that contribute the most to intakes of total sugar or added sugars/NMES. Fruits and vegetables contribute more and beverages contribute less to adults’ intakes in total sugars relative to those of children, in the four countries where age comparisons are possible. Age appears to be less strongly associated with contributors to added sugars or NMES, although a trend might be identified that adults obtain more of their added sugars from sweet products than do children, with the opposite being seen for beverages.

Globally, and independently of age, gender and countries, four food groups contribute to more than 85% of intakes in total sugars, which are sweet products, beverages, fruits and vegetables and dairy products. Sweet products and beverages provide more than two-thirds of added sugars or NMES, while dairy products contributes to 11 to 14% of added sugars and to 5% of NMES. Of relevance is the fact that products in these different food categories present different nutritional densities and thus do not have the same role in the diet. For example, cakes, pastries and sweets, and even more so soft drinks, usually provide low amounts of minerals, vitamins and fiber, and their favorable contribution to the supply in shortfall nutrients is limited, while dairy products are key contributors to calcium intake, and pure fruit juices are significant contributors to vitamin C intakes. In French children, for example, the sweet products category contributes to 48% of added sugar intakes and 16% of the intake of fiber, and sweetened beverages provide 14.4% of added sugars with no significant supply in any shortfall nutrient. By contrast, fresh dairy products provide 11.4% of the calcium supply and 8.3% of the added sugars (re-analyzed from [25]). In the UK, pure fruit juices, which contain no added sugars, but which are accounted for in the NMES estimate, contribute to 18% of the vitamin C supply in children aged 11 to 18 and to 11% of their NMES intake [34]. This should be taken into account when defining policy measures intended to lower added sugar without lowering the dietary intake of desired nutrients.

Re-analyzed data from the Netherlands and France showed that a lower educational attainment was associated with a higher added sugar intake; however, this was only statistically significant in Dutch adults. For total sugar intake, no such trend was observed in either France or the Netherlands. In a previous publication of the Dutch Food Consumption Survey 2007–2010 also only minor differences in consumption of total, free, and added sugars across income levels were observed [21]. No other European studies were found studying the association between sugar intake and measures of socio-economic status. In the US., data from four surveys from 2003 to 2010 showed that adults with a lower income consumed more added sugars than higher-income adults; no trend was observed in children [44]. Another US study on data from the National Health Interview Survey 2005 showed that a lower family income and educational status were each independently associated with a higher added sugar intake [45]. Furthermore, both low-income adults and children had a higher intake of sugar-sweetened beverages than their high-income counterparts [46]. Thus, previous findings from the US show a clearer trend between added sugar intake and socio-economic status than in Europe. This may be explained by the fact that in the US, energy-dense foods that are high in added sugars, refined grains, and fats are less costly than foods with a high nutrient density [47] and these cost differences may be more pronounced than in Europe.

Strengths of this review are its European focus and the representativeness of the surveyed populations. It also addresses in detail the contribution of different food groups to sugar intakes and the importance of educational levels, two considerations of outmost importance when public health policies are foreseen. Limitations also exist, that should be taken into account when interpreting the results. It cannot be guaranteed that all existing studies have been identified; it is possible that some surveys, available only in national languages, have been missed and it is likely that raw data from studies informing on total sugar can be exploited further for added sugar intakes, but we had no access to the original information.

Survey methodologies differ one from each other, on important features and at most steps of the survey process. These differences concern sampling procedures, and thus representativeness, but also the dietary data collection (dietary recall on 2, 3, 4 or 7 days and frequency check in a single study) and its management (softwares and grouping of foods, food composition tables, adjustment for confounding factors) as well as the display of results which often consider different age or gender groups and can be expressed with various units (weight, % of daily energy, energy intake accounting or not for alcohol, etc.). A significant weakness comes from the lack of robustness when dealing with added sugars. Although attempts are currently being made to find specific biomarkers [48], there is today no analytical means to measure added sugars, which have to be estimated through different methods. These methods are open to interpretation, as they are subjective and require a number of assumptions to be made about the types and sources of sugars present in the food. This is especially true for composite and processed foods such as breakfast cereals and many others. Of note also is the lack of information pertaining to “free” sugar intake, despite the fact that this item is concerned in the WHO or UK recommendation [1, 13].

The development of systematic methodology, as attempted in Australia [49], would be welcomed in Europe. Indeed, the difference in the methods currently used to estimate added sugars can lead to inappropriate estimates. For example, in Denmark, all the sugar present in cakes, desserts or breakfast cereals was considered as added; this may have led to a slight overestimation of added sugars (e.g. not considering lactose in a milk-containing cake), when in other surveys, added sugar was determined at the ingredient level, after disaggregation of the recipe. These small differences should not be disregarded: the expected changes in added sugar intakes in real life and at population levels may not be much higher, especially during the initial steps of implementation of policy measures.

As methodological differences across surveys make it difficult to develop fact-based nutrition policies at the pan-national level, the European Food Safety Agency is currently implementing and promoting among the European Member States a common methodology for dietary surveys, which includes recommendations about sampling procedures, data collection and treatment as well as on overall quality assessment [50]. Today, more than 16 European countries have been undertaking surveys according to these guidelines and the first set of data should be available shortly. Although these harmonized procedures will bring a very significant improvement, the question will remain of a reliable and comparable way to consider added sugars and/or free sugars and to estimate their amount in the diet.

## Conclusion

The available information on intakes in total or added sugars and NMES in Europe suggests that total sugars contribute to 15 to 25% of energy supply in several European countries, among which 7.5 to 17% are added sugars or NMES, the highest figure always being for children or teens. While there is no recommended threshold of appropriate intake for total sugars, there are recommendations pertaining to free sugars, set by WHO at 10% and by the UK at 5% of the total energy intake [1, 13]. From the available data, a large proportion of the European population, especially, but not only children, appears to exceed the 10% threshold. A recent survey in 1630 European teens from eight different towns found that 94% of them obtained more than 10% of their daily energy from NMES [51]. Although it can be argued that there is currently no firm evidence that added sugar is more harmful than excess calories from any other food source [52], these findings justify that the EU, as are many individual countries, is working on public health policy measures intended to lower intakes of added sugars or NMES.

Reformulation of products containing added sugars can be seen as one of the most straightforward routes. Nevertheless this measure should be designed thoughtfully and should rather target foods and food groups for which a decrease in sugar content would efficiently diminish the sugar supply in the target population. Simulations performed in the UK clearly show that reducing by half the sugar content of soft drinks would reduce by 14.4% the sugar intake of teens aged 11 to 18, while a similar decrease in yoghurt and dairy desserts would reduce it by 1.2% only. Figures are different in children aged 4 to 10, with a 7.9 and 3.1% decrease in sugar intakes due to soft drink and yoghurt reformulations, respectively (see annex 5 of reference [13]). This points out the importance of having reliable and detailed data about current food and nutrient intakes, in order to enable tailored policies that fit the needs of the population. This information should be available on sugars and added sugars, as well as on other nutrients, to allow for a global evaluation of the foreseen policy measures, after a few years of implementation. Although our review has identified data in countries accounting for a significant proportion of the European population, information is still missing for a large proportion of European countries. In addition, the analysis and interpretation of the currently available surveys only provide a limited and heterogeneous knowledge of sugar intakes in Europe, which might hamper the implementation and monitoring of efficient policy measures.

## Abbreviations

FFQ: Food Frequency Questionnaire
NMES: Non-milk extrinsic sugar
WHO: World Health Organization
